# Expression of Concern: Purification, characterization, and hydrolysate analysis of dextranase from *Arthrobacter oxydans* G6-4B

**DOI:** 10.3389/fbioe.2024.1450316

**Published:** 2024-07-02

**Authors:** Frontiers Editorial Office

With this notice, Frontiers states its awareness of serious allegations of undisclosed conflicts of interest surrounding the article “Purification, Characterization, and Hydrolysate Analysis of Dextranase From Arthrobacter oxydans G6-4B” published on 10 February 2022. Our Research Integrity team, will conduct an investigation in full accordance with our procedures. The situation will be updated as soon as the investigation is complete.

**UPDATE:** This article has now been retracted. Please find the full retraction here: https://doi.org/10.3389/fbioe.2025.1702209

